# Active behavior of triple-negative breast cancer with adipose tissue invasion: a single center and retrospective review

**DOI:** 10.1186/s12885-021-08147-2

**Published:** 2021-04-20

**Authors:** Junzo Yamaguchi, Hiroki Moriuchi, Takashi Ueda, Yujo Kawashita, Takanori Hazeyama, Masaki Tateishi, Shigehisa Aoki, Kazuyoshi Uchihashi, Mikio Nakamura

**Affiliations:** 1 Department of Surgery, Fukuoka Seisyukai Hospital, Fukuoka, 811-2316 Japan; 2 Department of Surgery, National Hospital Organization, Saga Hospital, Saga, Japan; 3 Department of Pathology and Microbiology, Faculty of Medicine, Saga University, Saga, Japan; 4 Department of Pathology, National Hospital Organization, Saga Hospital, Saga, Japan; 5 Department of Urology (Chair of the Board of Directors), Seisyukai Social Medical Corporation, Fukuoka, Japan

**Keywords:** Triple-negative breast cancer, Luminal breast cancer, Adipose tissue invasion, Tumor microenvironment, Patient survival

## Abstract

**Background:**

Interactions between adipocyte and breast cancer (BC) cells have yet to be fully elucidated. Here we investigated the prognostic impact of marginal adipose tissue invasion in both luminal breast cancer (HR+/HER2-) and triple-negative breast cancer (TNBC) (HR−/HER2-).

**Methods:**

A total of 735 patients with early-stage invasive BC (1999–2014) were retrospectively registered. Median length of patient follow-up was 8.9 years. Survival curves were calculated using a Kaplan-Meier cumulative survival plot. The prognostic difference between two groups were assessed by the univariate Cox-proportional hazard regression model.

**Results:**

Patients with adipose tissue invasion (*n* = 614) had a significantly poorer prognosis than those without adipose tissue invasion (*n* = 121) in overall survival (OS) (hazard ratio, 2.1; 95% Confidence interval [CI], 1.1 to 4.0; *P* = 0.025). While a poorer prognosis was observed in TNBC (*n* = 137) than in luminal BC patients (*n* = 496) (hazard ratio, 0.45; 95% CI, 0.30 to 0.68, *P* < 0.001), this aggressive nature of TNBC was noted in node-positive disease (hazard ratio, 0.3; 95% CI, 0.18 to 0.5, *P* < 0.001) but not in node-negative disease (hazard ratio, 0.78; 95% CI, 0.39 to 1.55, *P* = 0.472), and also noted in adipose tissue invasion-positive patients (hazard ratio, 0.4; 95% CI, 0.26 to 0.6, *P* < 0.001) but not in adipose tissue invasion-negative patients (hazard ratio, 0.73; 95% CI, 0.16 to 3.24, *P* = 0.675). In addition, although patients suffering from TNBC with adipose tissue invasion had a poorer outcome than those without adipose tissue invasion (hazard ratio, 3.63; 95% CI, 1.11 to 11.84; *P* = 0.033), the difference was not observed in luminal BC (hazard ratio, 1.75; 95% CI, 0.64 to 4.82; *P* = 0.277).

**Conclusions:**

Adipose tissue invasion was correlated with poor survival in TNBC. Cancer cell invasion into local fat may be a first step on cancer progression and systemic disease in TNBC.

**Supplementary Information:**

The online version contains supplementary material available at 10.1186/s12885-021-08147-2.

## Background

Obesity is a risk factor for many cancers including breast cancer (BC), but little is known about the relationship between cancer-cell invasion into local fat and patient survival in BC. The tumor microenvironment is a heterogeneous population of cells consisting of the tumor cells as well as endogenous stromal cells, such as vascular endothelial cells, pericytes, fibroblasts, bone-marrow mesenchymal stromal cells, immune cells and adipocytes [1]. These stromal cells are recruited by cancer cells and promote cellular migration, tumor angiogenesis, proliferation, invasion, and metastasis, as well as drug resistance [2]. Adipocytes are especially abundant stromal partners in breast tissue [3], and BC cell invasion into local fat seems to increase metastatic potential in the animal model [4].

Triple-negative breast cancer (TNBC) accounts for roughly 10–20% [5, 6] of all BC cases. The disease is viewed universally as the most devastating form of BC because of its aggressive nature [7, 8]. However, since there are still no markers to predict the efficacy of chemotherapy and well-defined molecular targets remain a topic of investigation [9], current treatment options for TNBC commonly focus on cytotoxic chemotherapy. Although more aggressive chemotherapy has resulted in an improved prognosis, it usually leads to serious deterioration in quality of life.

On the other hand, we have observed in the clinical setting that TNBC patients do not present a uniformly dismal prognosis. We have also noticed a characteristic feature of survival outcome, that is, most of the recurrent disease occurs within a few years after surgery, and the recurrence risk rapidly decreases thereafter [6, 8]. Strikingly different from luminal BC, where more than half of recurrent disease occurs more than 5 years after surgery [10, 11], this finding indicates the existence of TNBC patients who have a good prognosis without systemic disease as well as those who have a poor survival with residual disease after adjuvant chemotherapy [12].

The present study aims to evaluate the significance of adipose tissue invasion on patient survival in luminal BC and TNBC. It is very important to explore the role of adipose tissue for cancer progression.

.

## Methods

### Patients

This retrospective study was approved by our institutional review board. We reviewed records from all patients with primary invasive carcinoma of the breast who underwent surgery between 1999 and 2014 at our institution (National Hospital Organization, Saga Hospital). Of the 858 patients, 123 were excluded for the following reasons: synchronous bilateral breast cancer (*N* = 28), metachronous bilateral breast cancer in the period (*N* = 9), clinically multifocal or multicentric cancers in the unilateral breast (*N* = 39), distant metastasis (*N* = 24), inflammatory carcinoma of the breast (*N* = 6), ipsilateral breast recurrence after breast-conserving surgery (*N* = 2), tissues inappropriate for histoloigical review due to preoperative neoadjuvant chemotherapy (*N* = 4), and patients who died within 1 year (*N* = 11). Thus, our final study population comprised 86% (735/858) of the total potential patients. All were women except one case. The patients were followed up until June 31, 2019, the median length of follow-up being 107 months (8.9 years) (range, 1–244 months) after surgery. Of the 735 patients in the current study, 472 (64%) had been included in our previous reports [13]. The 472 patients were also approved by our institutional review board. This study did not involve the use of personal identifying information and individuals or families in the case of the deceased are not identified from data because of consecutive numbers of patients.

### Survival analysis

The events used to determine the breast cancer-specific survival (BCSS) rate included death due to breast cancer. The overall survival (OS) rate included death due to breast cancer and other causes. The survival of the two groups in each analysis was compared in the entire group of patients and subgroups adjusted for clinical-pathological factors.

### Tissue preparation and histological analyses

The resected breast and lymph node tissues were fixed in 10% formalin and the breast tissues were cut into 5-mm-thick slices [13, 14]. Each paraffin-embedded block was cut into 4-μm-thick sections and stained with hematoxylin and eosin. For the histological review, information on pathological tumor size, lymph node involvement (pN0: no regional lymph node metastasis, pN1: metastasis in 1 to 3 axillary ipsilateral lymph node, pN2: metastasis in 4–9 ipsilateral axillary lymph node, pN3: metastasis in 10 or more ipsilateral axillary lymph node), lymphatic vessel invasion, histological grade, adipose tissue invasion, hormone receptors (HR) status, and human epidermal growth factor receptor 2 (HER2) status was obtained from the prospective reports by pathologists (SA and KU, with 19 and 15 years of experience, respectively). The histological parameters between 1999 and 2009 were obtained from retrospective reviews conducted during the previous study by pathologists blinded to the survival outcome [13].

### Definitions of adipose tissue and marginal adipose tissue invasion

**Fig. 1:**
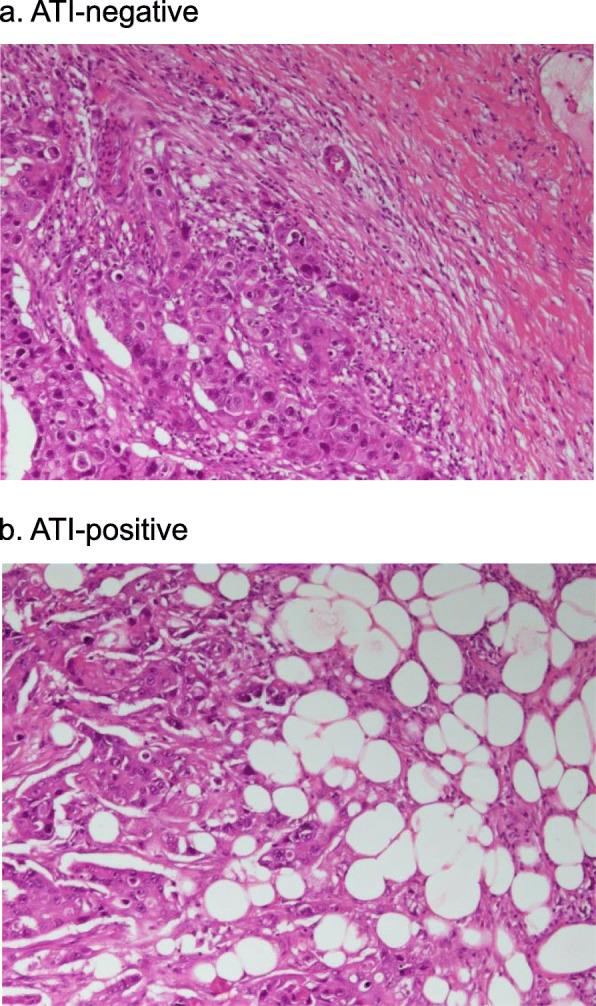
Histologic examination. Images show, **a**, cancer cells adjacent to a fibrous stroma devoid of adipocytes (adipose tissue invasion <ATI>-negative), and, **b**. cancer cells infiltrating directly into the surrounding adipose tissue (ATI-positive)

Histological sections were obtained from the whole cut surface of the tumor at the maximal diameter. The definition of adipose tissue and marginal adipose tissue invasion followed that in our previous reports [13, 14]. Adipose tissue was defined as a pure aggregate consisting of more than 20 fat cells without intervening fibrous tissues in the breast. The adipose tissue included tissues surrounding the mammary ducts or lobules, as well as those in the subcutaneous layers. Furthermore, fibrous tissue and fibroadipose tissue (fat cells mixed with various fibrous tissues) around the terminal duct-lobular unit were strictly differentiated from adipose tissues. Marginal adipose tissue invasion was defined as either the presence of more than 20 cancer cells in direct contact with the adipose tissue or as the presence of cancer cells in the adipose tissue. Pathologists reviewing breast cancers have routinely reported on the presence or absence of adipose tissue invasion in their clinical reports since 1999 (1999–2014). Representative images of negative and positive adipose tissue invasion are shown in Fig. 1a and b, respectively.

### Hormone receptor and HER2 status

Hormone receptor and HER2 status were evaluated as described previously [13–15]. Cases positive for estrogen receptor (ER) and progesterone receptor were defined as those with more than 10% of the cancer cell nuclei stained (ER-rich tumor). HER2 expression status was obtained on the basis of immunohistochemical analysis using commercially available antibodies. The intensity of HER2 staining was scored as follows: 0, 1+, 2+, or 3+. In the summary of results, we classified scores of 0 and 1+ as HER2 negative and scores of 3+ as HER2 positive. A HER2 score of 2+ was regarded as HER2-borderline and assessed by means of FISH assay.

### Statistical analysis

Univariate analysis was carried out by Student *t* test, and χ^2^ analysis. The prognostic difference between two groups (adipose tissue invasion-negative patients vs. adipose tissue invasion-positive patients and luminal BC vs. TNBC) were assessed by the univariate Cox-proportional hazard regression model. Survival curves were calculated using a Kaplan-Meier cumulative survival plot. Statistical analysis was conducted using the software StatView 5.0 for Mac (SAS Institute, Cary, NC). *P* < 0.05 was considered to indicate a statistically significant difference.

## Results

### Clinical-pathologic parameters for patients with and without adipose tissue invasion, and prognostic outcome (BCSS, OS)

Of the 735 cases, 614 (84%) and 121 (16%) were patients with and without adipose tissue invasion, respectively. We compared the two groups (Table 1). Patient age and body mass index were similar in the two groups. Tumor size was significantly larger and the frequency of nodal involvement significantly higher in patients with adipose tissue invasion (*P* < 0.001 in both). Stage I (pathological classification) was observed much more frequently in patients without adipose tissue invasion, while stage II or III was observed much more frequently in patients with adipose tissue invasion. (*P* < 0.001). The frequency of lymphatic vessel invasion was significantly higher in patients with adipose tissue invasion (*P* < 0.001). On the other hand, a low histological grade (grade I and II) was observed much more frequently in patients with adipose tissue invasion, and a high histological grade (grade III) was observed much more frequently in patients without adipose tissue invasion (*P* = 0.019). On the other hand, menopausal status was similar in the two groups.

**Table 1:** Comparison of patients with and patients without adipose tissue invasion (*n =* 735)

	ATI-negative	ATI-positive		
Clinical-pathologic features	(***N*** = 121)^a **b**^	(***N*** = 614)^a **c**^	***P*** Value	χ2^d^
age (y)^e^	58.0 ± 13.8 (26-86)	58.2 ± 13.1 (27-99)	0.872	…
Body mass index (kg/m^2^)^e^	22.8 ± 4.3 (13.6-37.7)	23.4 ± 3.8 (14.2-39.7)	0.105	…
Tumor size (cm)^e^	0.9 ± 0.7 (0.04-4.4)	2.1 ± 1.5 (0.1-13)	< 0.001	…
Lymph node involvement				
Negative	103 (89)	357 (59)	< 0.001	37.0 (1)
Positive	13 (11)	246 (41)		
Stage (pathological)				
I	97 (85)	265 (45)	< 0.001	63.6 (2)
II	14 (12)	220 (37)		
III	3 (3)	110 (18)		
Lymphatic vessel invasion				
Negative	108 (89)	385 (64)	< 0.001	29.3 (1)
Positive	13 (11)	215 (36)		
Histologic grade				
I, II (low)	64 (53)	388 (64)	0.019	5.5 (1)
III (high)	57 (47)	216 (36)		
Menopausal status				
Premenopausal	38 (32)	196 (33)	0.84	0.04 (1)
Postmenopausal	82 (68)	405 (67)		

The t-statistics for age, body mass index, and tumor size, respectively, were as follows; 0.16 (df, 733), 1.62 (df, 709), 8.6 (df, 724)

The 95% confidence intervals for age, body mass index, and tumor size were as follows: −2.37, 2.79; −0.13. 1.4; 0.91, 1.45

*ATI* adipose tissue invasion

^a^Except where indicated, data are number of patients, with percentage in parentheses

^b^Data are missing from five patients for lymph node involvement, seven patients for stage and one patient for menopausal status

^c^Data are missing from 11 patients for lymph node involvement, 19 patients for stage, 14 patients for lymphatic vessel invasion

10 patients for histologic grade, and 13 patients (including a man) for menopausal status

^d^Numbers in parentheses are the *df*

^e^Data are means ± standard deviations, with range in parentheses

We compared survival outcome between the two groups. The results of overall analysis (all) and subgroup analysis are shown in Table 2. A poorer prognosis was observed among patients with adipose tissue invasion (*N* = 609) than among those without adipose tissue invasion (*N* = 119) in breast cancer-specific survival (BCSS) (hazard ratio, 3.23; 95% confidence interval [CI], 1.31 to 7.95; *P* = 0.010) and overall survival (OS) (hazard ratio, 2.1; 95% CI, 1.1 to 4.0; *P* = 0.025) in the entire group of patients (all). Then, we adjusted for age (< 70 and > = 70), menopausal status (premenopausal and postmenopausal), lymph node involvement (negative and positive), pathological stage (I, II, III), and histologic grade (low grade and high grade) in OS. Patients with adipose tissue invasion showed a poorer rate of survival than those without adipose tissue invasion in the age of < 70 (hazard ratio, 2.69; 95% CI, 1.09 to 6.65; *P* = 0.032) and histologic high grade tumors (hazard ratio, 3.5; 95% CI, 1.27 to 9.67; *P* = 0.016).

**Table 2:** Comparison of survival (BCSS, OS) in patients with and without adipose tissue invasion

		ATI-negative	ATI-positive		
Survival	Patient groups	No of events/No of patients	No of events/No of patients	Hazard ratio	***P*** Value
		(%)	(%)	(95%CI)	
BCSS	All	5/119 (4)	89/609 (15)	3.23 (1.31-7.95)	0.01
OS	All	10/119 (8)	114/609 (19)	2.1 (1.1-4.0)	0.025
	Age				
	< 70	5/91 (5)	77/476 (16)	2.69 (1.09-6.65)	0.032
	> = 70	5/28 (18)	37/133 (28)	1.58 (0.62-4.04)	0.337
	Menopausal status				
	Premenopausal	1/38 (3)	28/196 (14)	5.32 (0.72-39.08)	0.101
	Postmenopausal	9/80 (11)	81/401 (20)	1.66 (0.83-3.31)	0.149
	Lymph node involvement				
	Negative	8/101 (8)	41/355 (12)	1.37 (0.64-2.91)	0.423
	Positive	1/13 (8)	71/243 (29)	3.66 (0.51-26.34)	0.198
	Stage				
	I	8/95 (8)	25/265 (9)	1.07 (0.48-2.37)	0.874
	II	1/14 (7)	36/217 (17)	1.61 (0.22-11.78)	0.64
	III	0/3 (0)	49/108 (45)	–	–
	Histologic grade				
	I, II	6/62 (10)	58/387 (15)	1.45 (0.62-3.36)	0.39
	III	4/57 (7)	54/212 (25)	3.5 (1.27-9.67)	0.016

*BCSS* breast cancer-specific survival, *OS* overall survival

### Clinical-pathologic parameters and treatment in luminal BC and TNBC

Of the 735 cases, 502 (68%) were luminal BC (estrogen and progesterone receptors positive/human epidermal growth factor receptor type 2 negative; HR+/HER2-), 137 (19%) were TNBC (HR−/HER2-) and 59 (8%) were HER2-enriched BC (HR−/HER2+). The remaining 37 cases consisted of 29 HR+/HER2+ breast cancers (4%) and 8 unknown cases. Since the number of HER2-enriched BC cases was small and adjuvant one-year treatment with trastuzumab had been administered since 2009 (1999–2014), we compared the two tumor subtypes in this study, that is luminal BC and TNBC.

First, we compared clinical-pathological parameters between the two subtypes (Table 3). No difference was observed in either patient age (*P* = 0.429) or body mass index (*P* = 0.233). A statistically significant difference was observed in the pathologic tumor size (*P* = 0.008), but the frequency of lymph node involvement, stage and lymphatic vessel invasion was similar (*P* = 0.832, *P* = 0.403 and *P* = 0.259, respectively). Luminal BC was associated with a lower histological grade, while TNBC were associated with a higher histological grade (*P* < 0.001). With regard to adipose tissue invasion, the frequency of masses with adipose tissue invasion was higher in luminal BC cases, while the frequency of masses without adipose tissue invasion was higher in TNBC cases (*P* < 0.001).

**Table 3:** Clinical-pathologic parameters in luminal BC TNBC, and treatment in those

	Luminal BC	TNBC		
	(***N*** = 502)^a b^	(***N*** = 137)^a c^	***P*** Value	χ2^d^
**Clinical-Pathologic Features**				
age (y)^e^	58.4 ± 13.4 (29-99)	59.4 ± 13.0 (26-89)	0.429	…
Body mass index (kg/m^2^)^e^	23.3 ± 3.9 (14.2-39.7)	22.9 ± 3.9 (13.6-37.8)	0.233	…
Tumor size (cm)^e^	1.9 ± 1.3 (0.04-10)	2.2 ± 1.9 (0.1-13)	0.008	…
Lymph node involvement				
Negative	318 (64)	84 (65)	0.832	0.05 (1)
Positive	178 (36)	45 (35)		
Stage (pathological)				
I	259 (53)	61 (48)	0.403	1.82 (2)
II	164 (33)	43 (34)		
III	67 (14)	23 (18)		
Lymphatic vessel invasion				
Negative	336 (68)	96 (73)	0.259	1.27 (1)
Positive	161 (32)	36 (27)		
Histologic grade				
I, II	378 (76)	48 (36)	< 0.001	76.53 (1)
III	121 (24)	86 (64)		
Adipose tissue invasion				
Negative	56 (11)	35 (26)	< 0.001	18.25 (1)
Positive	446 (89)	102 (74)		
**Treatment**				
Surgery for breast				
Bt	285 (57)	78 (57)	0.973	0.001 (1)
Bp	217 (43)	59 (43)		
Surgery for axillary lymph node				
ALND	229 (46)	76 (55)	0.002	15.05 (3)
sampling procedure	189 (38)	33 (24)		
SNB^f^	79 (16)	22 (16)		
nil	5 (1)	6 (4)		
Endocrine therapy	487 (97)	32 (23)	< 0.001	382.80 (1)
Chemotherapy				
intravenous	218 (43)	103 (75)	< 0.001	56.69 (2)
oral only^g^	19 (4)	11 (8)		
nil	265 (53)	23 (17)		
Radiation therapy				
PMRT	23 (5)	6 (4)	0.902	0.21 (2)
post conservative surgery	182 (36)	47 (34)		
nil	297 (59)	84 (61)		

The t-statistics for age, body mass index, and tumor size, respectively, were as follows; −0.79 (df, 637), 1.19 (df, 619), −2.67 (df, 630)

*Luminal BC* luminal breast cancer, *TNBC* triple-negative breast cancer

^a^Except where indicated, data are number of patients, with percentage in parentheses

^b^Data are missing from six patients for lymph node involvement, 12 patients for stage, five patients for lymphatic vessel invasion

and three patients for histologic grade

^c^Data are missing from eight patients for lymph node involvement, 10 patients for stage, five patients for lymphatic vessel invasion

and three patients for histologic grade

^d^Numbers in parentheses are the *df*

^e^Data are means ± standard deviations, with range in parentheses

**Treatment**

*ALND* axillary lymph node dissection, *SNB* sentinel node biopsy, *PMRT* postmastectomy radiation therapy

^f^SNB had been conducted since 2010 (1999-2014)

^g^Oral uracil and tegafur were selected

Furthermore, we outlined the treatment administered for luminal BC and TNBC patients (Continued). No difference was observed among cases undergoing breast surgery, but the frequency of axillary lymph node dissection (ALND) was higher in TNBC patients (*P* = 0.002). Endocrine therapy was performed for 97% of luminal BC patients and for 23% of TNBC patients (*P* < 0.001). Endocrine therapy was indicated for tumors in which more than 1% of the cancer cell nuclei were stained. On the other hand, intravenous routine adjuvant chemotherapy (doxorubicin-cyclophosphamide, docetaxel-cyclophosphamide, epirubicin- cyclophosphamide, epirubicin-cyclophosphamide followed by paclitaxel, fluorouracil-epirubicin-cyclophosphamide followed by docetaxel, fluorouracil-doxorubicin-cyclophosphamide, docetaxel followed by epirubicin-cyclophosphamide) (ASCO Clinical Practice Guideline 2018 [16]) was selected for 43% of luminal BC patients and for 75% of TNBC patients (*P* < 0.001). The median cycle of intravenous chemotherapy was four in luminal BC and six in TNBC. We usually establish the chemotherapy regimen (including no treatment) on the basis of the attending physician’s judgment, with reference to tumor size, lymph node status, overall performance status, presence or absence of medical comorbidities, and patient age. With regard to radiation therapy, no difference was observed between the two groups.

### Comparison of survival outcome (OS) between luminal BC and TNBC

**Fig. 2:**
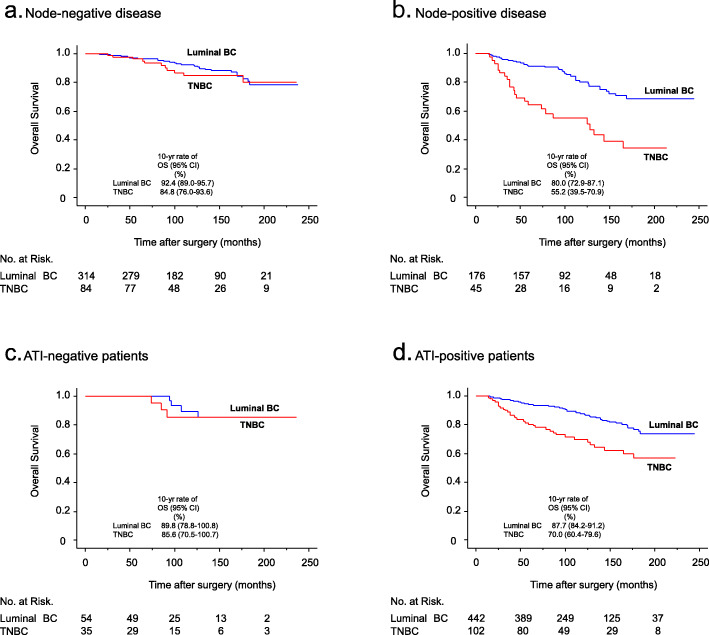
Comparison of survival outcome (OS) between luminal BC and TNBC in nodal status (**a**, **b**) and adipose tissue invasion (ATI) status (**c**, **d**). Graphs show, the Kaplan-Meier Plot of survival in node-negative disease (**a**), node-positive disease (**b**), ATI-negative patients (**c**), and ATI-positive patients (**d**). **a** *P* = 0.472, **b** *P* < 0.001, **c** *P* = 0.675, **d** *P* < 0.001

Next, we compared survival outcome between the two groups. The results of overall analysis and subgroup analysis are shown in Table 4. A poorer prognosis was observed among TNBC patients (*n* = 137) than among luminal BC patients (*n* = 496) in OS (hazard ratio, 0.45; 95% CI, 0.30 to 0.68, *P* < 0.001) in the entire group of patients (all). In subgroup analyses, a significantly poor outcome of TNBC was observed in the age (< 70 and > = 70) (*P* = 0.006 and *P* = 0.001, respectively), tumor size (<= 2 cm and > 2 cm) (*P* = 0.005 and *P* = 0.016, respectively). On the other hand, no difference was observed in node-negative disease (hazard ratio, 0.78; 95% CI, 0.39 to 1.55, *P* = 0.472) (luminal BC and TNBC were 314 and 84 cases, respectively), but a marked difference was observed in node-positive disease (hazard ratio, 0.3; 95% CI, 0.18 to 0.5, *P* < 0.001) (176 and 45 cases, respectively) (also shown in Fig. 2a and b). With regard to stage, no difference was seen in stage I, but a significant difference was noted in stage II (*P* = 0.002) and III (*P* = 0.012). In addition, a poorer prognosis was observed among TNBC patients than among luminal BC patients in histologic low grade tumors (hazard ratio, 0.32; 95% CI, 0.18 to 0.59, *P* < 0.001), but the prognosis was similar in high grade tumors (*P* = 0.23). Moreover, although no difference was observed in adipose tissue invasion-negative patients (hazard ratio, 0.73; 95% CI, 0.16 to 3.24, *P* = 0.675) (luminal BC and TNBC were 54 and 35 cases, respectively), there was a remarkable difference in adipose tissue invasion-positive patients (hazard ratio, 0.4; 95% CI, 0.26 to 0.6, *P* < 0.001) (those were 442 and 102 cases, respectively) (Fig. 2c and d).

**Table 4:** Comparison of survival (OS) in patients with luminal BC and patients with TNBC

	Luminal BC	TNBC		
Patient groups	No of events/No of patients	No of events/No of patients	Hazard ratio	***P*** Value
	(%)	(%)	(95%CI)	
All	66/496 (13)	36/137 (26)	0.45 (0.3-0.68)	< 0.001
Age				
< 70	41/380 (11)	22/106 (21)	0.48 (0.29-0.81)	0.006
> = 70	25/116 (22)	14/31 (45)	0.34 (0.17-0.65)	0.001
Tumor size (cm)				
< = 2	36/346 (10)	18/84 (21)	0.44 (0.25-0.78)	0.005
> 2	30/146 (21)	18/50 (36)	0.49 (0.27-0.87)	0.016
Lymph node involvement^a^				
Negative	31/314 (10)	11/84 (13)	0.78 (0.39-1.55)	0.472
Positive	35/176 (20)	23/45 (51)	0.3 (0.18-0.5)	< 0.001
Stage				
I	22/257 (9)	7/61 (11)	0.78 (0.33-1.82)	0.563
II	18/161 (11)	13/43 (30)	0.32 (0.16-0.65)	0.002
III	24/66 (36)	14/23 (61)	0.43 (0.22-0.83)	0.012
Histologic grade				
I, II	44/375 (12)	14/48 (29)	0.32 (0.18-0.59)	< 0.001
III	22/118 (19)	22/86 (26)	0.7 (0.39-1.26)	0.23
Adipose tissue invasion^b^				
Negative	4/54 (7)	3/35 (9)	0.73 (0.16-3.24)	0.675
Positive	62/442 (14)	33/102 (32)	0.4 (0.26-0.6)	< 0.001

^a^Refer to Fig. 2a and b

^b^Refer to Fig. 2c and d

### Prognostic outcome for patients with and without adipose tissue invasion in luminal BC and TNBC subtypes

We determined the prognosis of patients with and without adipose tissue invasion in the tumor subtypes. As shown in Table 5, no difference was observed in BCSS (hazard ratio, 1.75; 95% CI, 0.55 to 5.63; *P* = 0.346) or OS (hazard ratio, 1.75; 95% CI, 0.64 to 4.82; *P* = 0.277) when compared the cases of luminal BC with and without adipose tissue invasion (adipose tissue invasion-negative and adipose tissue invasion-positive cases were 54 and 442, respectively). On the other hand, we compared cases of TNBC with and without adipose tissue invasion. A significant differences was observed between the two groups in both BCSS (hazard ratio, 8.63; 95% CI, 1.17 to 63.66; *P* = 0.035) and OS (hazard ratio, 3.63; 95% CI, 1.11 to 11.84; *P* = 0.033) (those were 35 and 102, respectively). In TNBC, one of the patients without adipose tissue invasion died due to breast cancer diagnosed as node-positive disease at surgery.

**Table 5:** Survival (BCSS, OS) for patients with and without ATI in luminal BC and TNBC

		ATI-negative	ATI-positive		
Subtype	Survival	No of events/No of patients	No of events/No of patients	Hazard ratio	***P*** Value
		(%)	(%)	(95%CI)	
Luminal BC	BCSS	3/54 (6)	47/442 (11)	1.75 (0.55-5.63)	0.346
	OS	4/54 (7)^a^	62/442 (14)^a^	1.75 (0.64-4.82)	0.277
TNBC	BCSS	1/35 (3)	26/102 (25)	8.63 (1.17-63.66)	0.035
	OS	3/35 (9)^a^	33/102 (32)^a^	3.63 (1.11-11.84)	0.033

*BCSS* breast cancer-specific survival, *OS* overall survival

^a^Data were also shown in Table 4

## Discussion

Our study design focused on two tumor subtypes of BC (HR+/HER2- and HR−/HER2-) and defined cases with ER-rich tumors (more than 10% of the cancer cell nuclei stained) as hormone receptor-positive carcinoma. Furthermore, although this study is a single center review, we have made a sharp distinction between adipose tissue invasion-negative and adipose tissue invasion-positive BC. As a result, we obtained three valuable findings. First, tumors with adipose tissue invasion had a poorer prognosis than those without adipose tissue invasion in OS, as observed in high grade tumors. Second, although, as might be anticipated, TNBC showed a poorer survival than luminal BC, it was remarkable to find this to be the case in node-positive disease as well as adipose tissue invasion-positive patients. Third, patients suffering from TNBC with adipose tissue invasion had a poorer outcome than those without adipose tissue invasion, in contrast to luminal BC cases. Namely, it is likely that a poorer outcome of tumors with adipose tissue invasion was due to the cases of TNBC.

We reported previously that patients with adipose tissue invasion showed a poorer disease-free survival (DFS) than those without adipose tissue invasion [13, 14]. In the present study, the poor outcome was observed both in breast cancer-specific survival (BCSS) and, importantly, in OS. In addition, the results were found in TNBC patients. Marginal adipose tissue invasion resulted in marked cell changes in the course of tumor activity in TNBC. When we compared TNBC to luminal BC, we found that the poor survival of TNBC was seen in node-positive disease but not in node-negative disease and, therefore, that the highly aggressive nature of TNBC was due to lymph-node metastasis but not due to the tumor size or histological tumor grade in this study. Since adipose tissue invasion independently affected the nodal involvement in our previous study [14], it is suggested that the active behavior of adipose tissue invasion-positive TNBC is related to lymph-node metastasis.

Currently, the literature is scattered with studies on adipocyte biology in BC [1, 3]. Indeed, adipocytes are considered to constitute a critical cell type in the tumor microenvironment of BC [17, 18]. Recently, moreover, some investigators highlighted the striking effects of adipocytes on the human TNBC cell line, that is, enhanced cell migration and invasion [19]. In the present study, it is suggested that nodal status is a very strong prognostic factor in TNBC. A relationship may exist between marginal adipose tissue invasion and the involvement of the functional lymphatic endothelium.

Meanwhile, a thoroughgoing estimation of a patient’s prognosis is crucial to avoid overtreatment and biological understanding helps us to escalate and de-escalate therapy even in high risk tumors [20]. In TNBC, few patients suffer recurrence between 5 and 10 years after surgery, and it is unlikely that chemotherapy exerts any major impact on late recurrence [21]. We think that there is a group of patients whose disease is localized at the time of diagnosis. As shown in the results, of TNBC without adipose tissue invasion (*n* = 35) BC related-death was only one case. Although the confidence interval was very broad and the risk was unstable, most of adipose tissue invasion-negative TNBC seem to be localized disease. With regard to adjuvant management strategies, it is conceivable that the absolute benefit of chemotherapy is relatively small in those cases.

Tumors are composed of both cancer stem-like cells and other differentiated cancer cells [22]. It has been proposed that the cancer stem-cell theory provides an insight into the aggressive nature of TNBC [12]. The TNBC phenotypes are highly similar to the cancer stem-cell phenotypes responsible for cancer progression, lymph node metastasis and distant metastasis as well as tumor initiation [12, 23]. In addition, accumulating evidence indicates that epithelial to mesenchymal transition shows similarities between the TNBC and cancer stem-cell phenotypes. It was reported that adipocytes from visceral white adipose tissue exert an enhanced effect on the epithelial to mesenchymal transition of BC cells [24]. There may be some relationship between cancer cell invasion into adipose tissue and expanding breast cancer stem cells in TNBC. Chemotherapy can eliminate the bulk of differentiated cancer cells but fail to eliminate breast cancer stem cells [25].

The present study is limited in that it is an observational retrospective study, and it is relatively small in scale for an analysis of prognosis especially for evaluating patients without adipose tissue invasion. Needless to say that triple negative breast cancers are heterogenous and encompass tumors with different histopathological features, but we have not been taken into account and classified TNBC cases with hormone receptor and HER2 status in this study. Moreover, tumor infiltrating lymphocytes (TILs) have been shown to exhibit a good prognosis in histologic screening, particularly in TNBC [26]. This might be useful for clinical decisions as a prognostic marker and should be included in pathology reports, but we have no data on the assessment of TILs.

## Conclusions

In conclusion, cancer-cell invasion into local fat seems to change a tumor from “a silent bystander to an active facilitator [17]” in TNBC. The next question is: Does adipose tissue invasion signal the start of the acquirement of malignant properties or the start of systemic disease in TNBC tumor cells?

## Supplementary Information

**Additional file 1.**

## Data Availability

All data (except for treatment data) generated or analysed for the current study are included in this supplementary information files. Treatment datasets will be available from the corresponding author on reasonable request.

## Abbreviations

BC: Breast cancer
BCSS: Breast cancer-specific survival
CI: Confidence interval
ER: Estrogen receptor
HR: Hormone receptors
HER2: Human epidermal growth factor receptor 2
OS: Overall survival
TNBC: Triple-negative breast cancer
